# Clinical effectiveness of posterior-only approach using polyetheretherketone cage combined with single-segment instrumentation for lumbar tuberculosis in children

**DOI:** 10.1038/s41598-021-03029-w

**Published:** 2021-12-06

**Authors:** Zhengquan Xu, Lanhua Chen, Changsheng Wang, Liqun Zhang, Weihong Xu

**Affiliations:** 1 Department of Spine Surgery, The First Affiliated Hospital of Fujian Medical University, Fuzhou, 350005 Fujian People’s Republic of China; 2 Trauma Medical Center of Fujian Province, Fuzhou, 350005 Fujian People’s Republic of China; 3 Department of Emergency, The First Affiliated Hospital of Fujian Medical University, Fuzhou, 350005 Fujian People’s Republic of China

**Keywords:** Infectious diseases, Neurological disorders, Psychiatric disorders

## Abstract

We sought to investigate the outcomes of posterior-only approach using polyetheretherketone (PEEK) cage combined with single-segment instrumentation (modified-approach) for mono-segment lumbar tuberculosis in children. Between February 2008 and August 2017 in our hospital, 18 children with single-segment lumbar tuberculosis enrolled in this study were treated by modified-approach. Medical records and radiographs were retrospectively analyzed. Mean follow-up time was 54.6 ± 12.1 months. No severe complications were noted to have occurred. Measures indicated there was satisfactory bone fusion for all patients. Mean Cobb angles were significantly decreased from preoperative angle (19.8° ± 13.1°) to those both postoperatively (− 4.9° ± 7.6°) and at final follow-up (− 3.5° ± 7.3°) (both P < 0.05), with a mean angle loss of 1.7° ± 0.9°. The erythrocyte sedimentation rate (ESR) returned to normal levels for all patients within 3 months postoperatively. All patients had significant postoperative improvement in neurological performance. The modified-approach was an effective and feasible treatment option for mono-segment children with lumbar tuberculosis. Such procedures can likely help patients by increasing retainment of lumbar mobility and reducing invasiveness.

## Introduction

Spinal tuberculosis (TB), accounts for about 50% of skeletal related cases of TB, and has been rising steadily at a global scale, especially in undeveloped areas^1,2^. Due to risk factors that can be relatively common including such as a weak immune system, malnutrition, and human immunodeficiency virus, children that might suffer from these circumstances are also more likely to be attacked by spinal TB^3^. As pediatric spinal TB make up a relatively high proportion of spinal TB and have unique characteristics involved with dynamics of growth and development of children, this health concern has rightfully demanded increasing priority in TB related health and research programs through nationally^4^.

Pediatric lumbar TB is one such type of an affliction, which needs to be treated cautiously because of anatomical characteristics and vertebral growth of child-aged patients^5^. Although anti-TB based chemotherapies play a critical role in the treatment of spinal TB, progressive kyphosis often still occurs even after clinical healing when only conservative treatments are used. Therefore, for patients afflicted with lumbar TB and with corresponding degrees of spinal instability, deformity, neurologic dysfunction, and abscesses, surgically-based treatments have typically been necessary. In recent years, some scholars have advocated a posterior-only approach as this approach could also be an effective treatment method option for adult patients with spinal related TB afflictions and is accompanied by minor trauma than other more traditionally used methods^6,7^. However, because of anatomical characteristics and growth of the spine of children-aged patients, the decision of whether to choose a treatment founded upon long- or short- segment fixation is controversial and difficult^8^. Debates regarding the optimal choices for bone grafting materials, including autograft, allograft, and PEEK and titanium mesh cages, are also ongoing. PEEK cage combined with single-segment instrumentation used on child patients with mono-segment lumbar tuberculosis was not reported. Therefore, the purpose of our study was to evaluate the feasibility and efficacy of debridement, and reconstruction using PEEK cage combined with single-segment instrumentation via a posterior-only approach for the treatment of mono-segment children afflicted with lumbar TB.

## Methods

### Basic information

A total of 18 pediatric-aged mono-segment lumbar TB patients who were treated via a posterior-only approach using PEEK cage combined with single-segment instrumentation were enrolled in the study. Clinical and experimental data were recorded and retrospectively reviewed in this study from a period spanning February, 2008 through August, 2017 at our hospital and treatment center. There were eight females and ten males with an average age of 10.5 (range 6–15) years (Table 1) at the initiation of surgery. All cases were diagnosed as having only one unit of spinal function involved in dysfunction (i.e., two adjacent vertebrae and the intervening disc). Along with this precondition, patients enrolled in this study also met the following conditions: (1) case with only mono-segment lumbar TB or simple vertebral TB; (2) patient with relatively intact pedicles of affected vertebrae without invasion of TB and which can facilitate strong anti-pull-out strengths of pedicle screws; (3) case without severe kyphosis which otherwise requires long-segment fixation plus osteotomy; (4) limited paravertebral or epidural abscess. Such included patients were noted to have accompanying neurological disorders or spinal instability which required surgical intervention. Exclusion criteria included severe kyphosis deformity, invasion of the pedicle of vertebra by TB, huge paravertebral abscess or psoas abscess, or any other serious multilevel spinal TB. Such excluded cases needed posterior long-segment instrumentation or combined anterior and posterior approaches. Written informed consent for participation in the study was obtained from parent or their guardian. All parents or their guardian knew the possible risks of PEEK cage. And the study strictly abided by the CARE Guidelines. The First Affiliated Hospital of Fujian Medical University Ethics Committee reviewed and approved the study protocol.

**Table 1:** Clinical data of patients and outcome.

Case no	Age (years)	Gender	Level	Operative time (min)	Blood loss (ml)	ESR (mm/h)		Follow-up time (months)
						Pre-op	TMP*	
1	6	F	L4–5	110	330	65	11	56
2	12	M	L3–4	90	250	43	8	49
3	14	M	L4–5	105	285	44	6	44
4	11	M	L1–2	75	200	74	14	63
5	7	F	L3–4	115	320	28	5	71
6	15	M	L2–3	85	265	53	10	42
7	9	M	L3–4	80	240	38	11	39
8	8	M	L4–5	100	295	31	12	54
9	11	F	L3–4	85	300	42	5	74
10	13	M	L1–3	110	310	57	3	48
11	8	F	L5–S1	85	265	81	9	56
12	9	M	L2–3	80	250	49	7	84
13	12	F	L4–5	90	275	62	10	62
14	14	F	L2–3	100	300	48	9	46
15	11	M	L2–4	140	235	75	11	52
16	9	F	L4–5	115	320	64	13	58
17	10	F	L1–2	95	360	58	6	41
18	15	M	L3–4	130	245	46	12	44
Mean	10.5 ± 2.4			99.4 ± 17.5	280.3 ± 39.1	53.2 ± 14.6	9.0 ± 3.0	54.6 ± 12.1

*Pre-op* pre-operative, *TMP* three months post-operative.

*Compared with pre-operative value, *P* < 0.05.

Children afflicted with mono-segment lumbar TB in this study were observed to have varied symptoms which included back pain, anorexia, weakness, muscular spasms, fever with sweats, weight loss, lower extremity radiation pain, or decreased spine mobility. No patients were with diagnoses of active lung TB or HIV positive. Diagnoses were confirmed according to non-specific laboratory tests in close combination with image-based findings such as spinal radiographic films, CT, and MRI. Based upon measures for the ASIA impairment scale, four cases were classified as having a grade of severity of C, six as grade D, and eight as grade E (Table 2). Preoperative and final follow-up measures of patient pain were evaluated by VAS. The Cobb angle was measured for laterally oriented spinal radiographic films.

**Table 2:** Radiological examination and neurological function of patients.

Case no	Cobb angle (°)					ASIA			VAS	
	Pre-op	Post-op*	FFU^#^	Correction	LC	Pre-op	Post-op	FFU	Pre-op	FFU**
1	31	4	6	27	2	D	E	E	6	1
2	2	− 10	− 10	12	0	E	E	E	4	0
3	27	− 4	− 2	31	2	D	E	E	6	2
4	32	1	3	31	2	C	D	E	8	1
5	16	− 11	− 10	27	1	E	E	E	5	1
6	28	4	5	24	1	E	E	E	7	0
7	21	− 8	− 6	29	2	D	E	E	6	0
8	29	− 7	− 4	36	3	E	E	E	4	1
9	34	6	8	28	2	C	D	E	7	1
10	− 6	− 16	− 15	10	1	E	E	E	5	2
11	33	3	4	30	1	C	C	D	7	1
12	− 2	− 20	− 17	22	3	E	E	E	6	1
13	32	− 3	− 2	29	1	D	E	E	8	0
14	26	− 6	− 5	32	1	D	E	E	8	2
15	12	− 2	− 4	14	2	E	E	E	5	1
16	− 2	− 18	− 15	16	3	E	E	E	7	1
17	16	1	0	15	1	C	D	E	5	0
18	18	− 2	1	20	3	D	E	E	5	0
Mean	19.8 ± 13.1	− 4.9 ± 7.6	− 3.5 ± 7.3	24.1 ± 7.6	1.7 ± 0.9				6.1 ± 1.3	0.9 ± 0.7

*Pre-op* pre-operative, *Post-op* post-operative, *FFU* final follow-up, *LC* loss of correction.

*Compared with pre-operative value, *P* < 0.05; ^#^Compared with pre-operative value, *P* < 0.05;

**Compared with pre-operative value, *P* < 0.05.

### Preoperative procedure

Prior to surgery, regular anti-TB chemotherapy including components of isoniazid (H, 10 mg/kg/d), rifampicin, (R, 10 mg/kg/d) and pyrazinamide (Z, 25 mg/kg/d) was administered for average 2–4 weeks for all patients depending upon drug dosing. Operations were then conducted when ESR was found to have obviously decreased and the general condition of the patient had recovered.

### Surgical method

Patients were placed in a prone position and were under general endotracheal anesthesia. A posterior midline incision was made, and a subperiosteal dissection of the affected vertebrae was performed. Pedicle screws were installed in the affected vertebrae, and in some cases, we used short-length pedicle screws with fixation based upon considerations of remaining vertebral body heights after debridement. The mild side of the lesion was stabilized by a temporary rod to avoid nerve injury during debridement. Then, the worse side of the lesion was chosen. Partial laminae and articular processes were resected in order to facilitate exposure of the affected vertebral body. Lesions including sequestra, abscesses, and granulomatous tissues, were debrided by way of using various curvature curettes under direct vision (rotating the operating table when necessary for purposes of operation). Pressurized washing was applied by way of inserting a catheter into the deep area and was performed in order to completely remove necrotic tissues and abscesses. Permanent rods were then placed and secured. Appropriate size PEEK cage filled with bone (healthy lamina, partial articular process, or allograft bone when necessary) were embedded into the interbody. Finally, drainage was performed and the incision was sutured. The operation was performed by the same group of surgeons. The PEEK cage was depuy synthes spine (USA).

### Postoperative procedure

During postoperative procedures, the drainage tube was removed when the fluid was less than 20 mL per 24 h. Nutritional support was enhanced. Anti-TB treatment was continued with the regimen of 3HRE/9–12HR postoperatively. Weight-bearing ambulation was started after lying in bed for 4 weeks postoperatively with the assistance of plastic orthosis. Regular follow-ups were performed and the value of hepatic function and ESR were regularly monitored.

### Follow-up evaluation and statistical analysis

During follow-ups, measures for indexes were recorded as follows: (1) neurological status, (2) Cobb angle, (3) loss of correction, and these were calculated as follows: final follow-up Cobb angle—postoperative Cobb angle, (4) ESR, (5) and the VAS pain score. Statistical analysis was managed by SPSS version22.0 (IBM Corp., Armonk, NY). Paired t-test was chosen to analyze changes of the indexes of Cobb angle, VAS score and ESR preoperatively, postoperatively, and during follow-up.

### Ethics declarations

Written informed consent for participation in the study was obtained from parent or their guardian. All parents or their guardian knew the possible risks of PEEK cage. And the study strictly abided by the CARE Guidelines. The First Affiliated Hospital of Fujian Medical University Ethics Committee reviewed and approved the study protocol.

### Consent to publish

All patients signed informed consent forms to publish their personal details in this article.

## Results

The average follow-up time was 54.6 months. Data for patients are presented in Table 1. Clinical symptoms for all children significantly improved postoperatively. Neurological status was found to have improved at varying degrees in all cases (Table 2). Measures of blood loss, operation time, and ESR were recorded and were listed in Table 1. The ESR returned to normal within 3 months postoperatively. Statistical comparisons of pre- and post-operative changes in VAS scores and ESR were found to have been statistically significant (P < 0.05).

**Figure 1:**
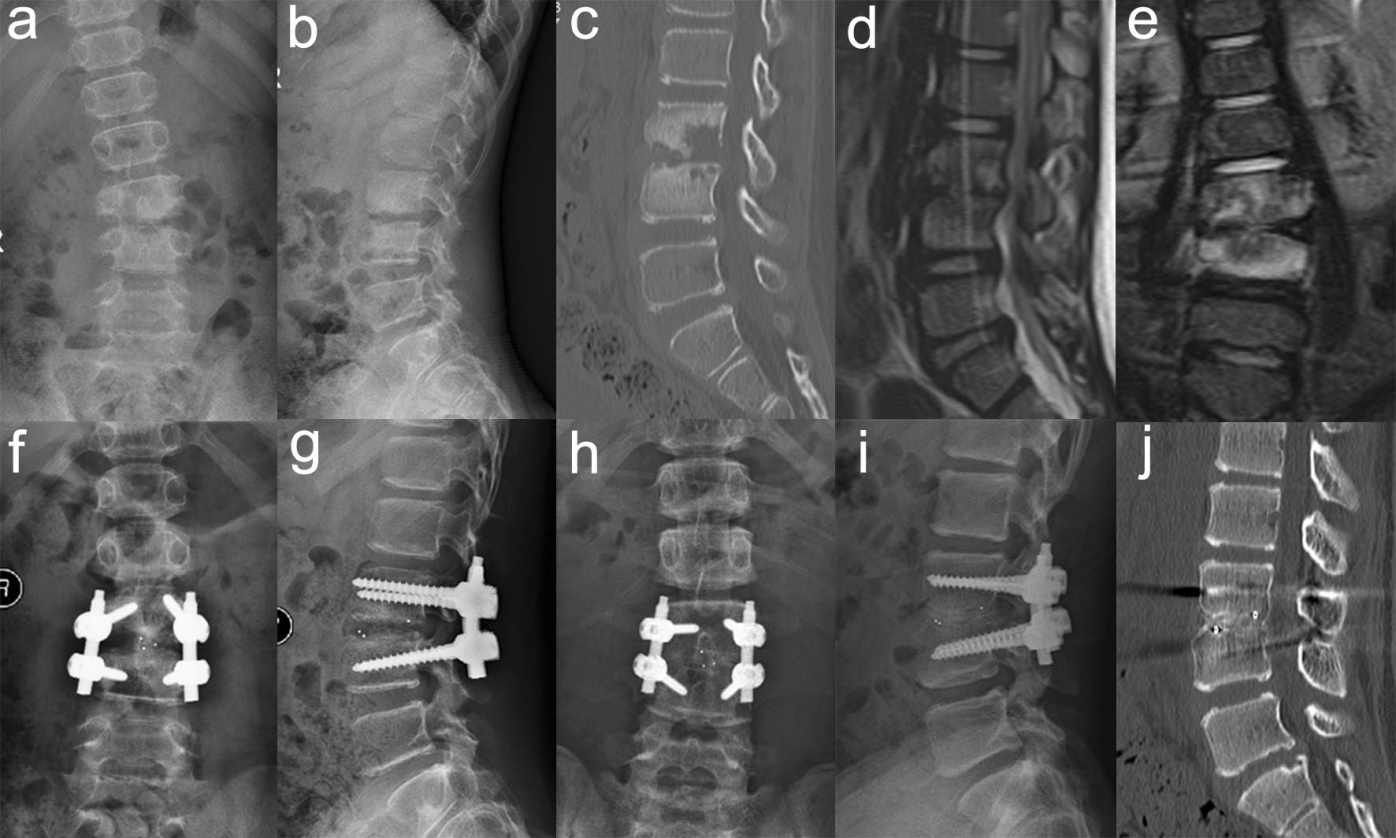
A 12-year-old boy with spinal tuberculosis in L3 to L4 underwent posterior-only approach using PEEK cage combined with single-segment instrumentation. (**a**–**e**) Preoperative images showing the collapse of vertebral body and imbalanced spinal growth. (**f**–**g**) Postoperative radiograph revealing good internal fixation and correction of imbalanced spine. (**h**–**j**) 49-month follow-up images depicting internal fixation in a good position and solid bone fusion.

Solid bone fusion was found in all patients though CT images showing the presence of bridging trabecular bone between the graft and host bone (Fig. 1). There were significant differences (P < 0.05) between pre- and post-operative, pre- and final follow-up measures for Cobb angle (Table 2). The mean correction of Cobb angle was 24.1° ± 7.6° postoperatively. And the average loss of correction was 1.7° ± 0.9° for observations made at final follow-up.

All patients underwent postoperative healing without complications such as wound infection, abscess or sinus formation, instrumentation or graft failure. Side effects of anti-TB drugs were found in one case whose hepatic dysfunction was observed 6 weeks after chemotherapy and was cured with the usage of liver-protecting agents.

## Discussion

Because of corresponding features of individuals with weakened immune systems, experiencing malnutrition, and human immunodeficiency virus, spinal TB in children account for a substantial portion of all diagnosed cases of spinal TB^3^. Spinal TB in children, whose fibrous rings and endplate cartilage are rich both in blood and lymphatic vessels, are more likely to propagate to different spinal segments than that in adult-aged patients^9^. Furthermore, spinal cords are nourished through smaller epidural spaces and blood vessels in pediatric-aged patients than in comparison to adults, resulting spinal TB afflictions in children with consequential higher related risks of neurological damage^10^. In addition, unbalance in the dynamics of spinal growth between anterior-middle columns, the most frequently involved site of spinal TB, and posterior columns, or heterogeneous types of injuries related to epiphysis of diseased vertebral bodies, makes pediatric-aged patients more prone to scoliosis or kyphosis compared to adult-aged patients. This is especially true when ages correspond to most rapid growth periods in child-aged patients. Accordingly, other research has identified specific types of biomechanical changes, which may also affect the morphology of deformities of spinal columns in children-aged patients, and can ultimately become types of a very harmful negative-feedback loop with impactful consequences^11–14^.

Anti-TB treatments have, and continue to play the key and cornerstone roles for treatment of patients afflicted by spinal TB. However, Rajasekaran et al.^15^ and Tuli et al*.*^16^ reported that some patients eventually developed severe deformities when they were treated conservatively. Therefore, surgical management of pediatric lumbar TB is urgently necessary for focus removal and kyphotic deformity correction in combination with chemotherapy^17,18^.

The treatment of lumbar TB in children-aged patients is in many ways similar to that in adults. A posterior-only approach offers opportunity to increase levels of safety, can be less invasive, and is a relatively easier operation to implement, and has become increasingly widely advocated through the development and implementation of pedicle screws, especially for children-aged patients. A posterior-only approach can effectively avoid potential complications related to complex anatomy of the retroperitoneal area and can help to reduce levels of risk of damages to large blood vessels and vital organs^8^. Furthermore, the posterior-only approach requires only a single incision, rather than two incisions typically required in combined anterior and posterior approach, thereby minimizing the scarring in children-aged patients and also reducing the pain caused by two incisions. In our study, we found that measures related to VAS significantly decreased from 6.1 ± 1.3 to 0.9 ± 0.7 by the last follow-up. Concurrently, lesions and abscesses of involved regions could be removed as efficiently and thoroughly as possible by way of using an angle of 270° and with the use various types of curvature curettes under naked eye by rotating the operating table. Moreover, this approach had the advantages of a relatively short operation time, small surgical trauma, and less blooding loss. These outcomes are in particularly important with respect to children-aged patients with correspondingly smaller blood volumes and poorer levels of tolerance to surgery than for comparisons with respect to adult-aged patients. In our study, the average blood loss was only 280.3 ± 39.1 ml, minimize the trauma to the children patients.

The ranges of fixation and fusion that should be applied for the treatment of spinal TB in children are points of considerable debate. Some experts have suggested that long-segment fixation is ideal, whereas other experts have advocated the use of short-segment fixation as the ideal choice. However, both technologies sacrifice at least two normal motion units of the spine and may produce or induce the development of adjacent vertebral diseases in later periods^19^. In addition, both can cause the posterior column of the normal vertebral body to stop growing due to the application of fixation with screws and rods while the anterior and middle columns contrastingly continue to grow for the existence of endplate cartilage. Such types of asymmetrical growth may lead to eventual spinal imbalances. Furthermore, subperiosteal dissection of joints and lamina of normal motion units is avoided through single-segment fixation, which might facilitate reductions in probabilities of spontaneous fusion of adjacent segments and thus limit corresponding interference with spinal growth^20^. Moreover, single-segment fixation mostly has the benefit of furthering patient retention of levels of lumbar mobility, thereby reducing impacts upon daily life. In the approach we used, screws were only inserted into the pedicles of affected vertebrae (we used short-length pedicle screws when necessary in some cases). Furthermore, all procedures, including debridement, decompression, and interbody fusion, were conducted in spaces confined only to the TB affected segments and were completed without any disruptions of normal motion units. After implantation of PEEK, the upper and lower pedicles were compressed in order to enhance the firmness of cage and correct spinal kyphosis. The mean correction of kyphotic angle was 24.1° ± 7.6°, which decreased from 19.8° ± 13.1° preoperatively to − 4.9° ± 7.6° postoperatively, and was effectively maintained with an average loss of 1.7° ± 0.9° at last follow-up (Fig. 1). Outcomes were similar to the results reported by surgeons who adopted long-segment fixation or combined anterior and posterior approach in the treatment of lumbar TB in children. Hu et al*.*^10^ reported that a correction angle of 25.2° was achieved by way of using long-segment fixation, and reported a corresponding correction loss of 1.1°. Zhang et al*.*^21^ pointed out findings, which indicated that the correction angle was 25.3° when patients were treated with the combined anterior and posterior approach, and reported a correction loss of 0.8°.

The choice of grafting material for use in interbody fusion after surgical debridement is another concern. In general, autologous bones such as autogenous rib and iliac crest are widely advocated and considered as the gold standard in bone defect management^22–24^. However, the sources of autogenous bone, often associated with significant donor site morbidities and more trauma, is limited for children^25^. Besides, graft-related failures may occur because of disruption, absorption, subsidence, or slippage, ultimately inducing failure of internal fixation devices. Scholars have confirmed that titanium mesh cages used in the management of spinal TB were secure without invalidity of antituberculotic effectiveness and occurrence of bacterial infection^21–23,26^. To our knowledge, no studies have recorded the clinical effectiveness of PEEK cage in the treatment of lumbar TB in children. However, literature has demonstrated that inertness and biocompatibility of PEEK cages were equivalent to titanium mesh cages^27,28^. Therefore, we undertook reconstruction of bone defects formed after debridement with PEEK cage that were filled with autogenous bone (healthy lamina, partial articular process), or allograft bone when necessary. The strength and rigidity bearing capacities of PEEK cage could provide ample and forceful support together in conjunction with the pedicle screws. Besides, PEEK cage has the characteristics of high friction on the contact surface between cage and vertebral body, less likely to prolapse of cage. Peek cage provides sufficient support for anterior column of vertebral body, which could restore the stability of spine and reduce the loss of correction angle to maximum extent. During follow-ups, no implant or fusion failures were found to occur and no recurrence of spinal TB was identified for all patients.

Each case of lumbar TB in children should be individually managed, because controversy remains over the best treatment options. When adopting such methods, strict operative indications should be emphasized: (1) case with only mono-segment lumbar TB or simple vertebral TB; (2) patient with relatively intact pedicles of affected vertebrae without invasion of TB and which can facilitate strong anti–pull-out strengths of pedicle screws; (3) case without severe kyphosis which otherwise requires long-segment fixation plus osteotomy; (4) limited paravertebral or epidural abscess.

Considering that the sources of autogenous bone is often associated with significant donor site morbidities and more trauma, and is limited for children, most lumbar TB in children in our hospital were treated with PEEK cage. Besides, the incidence of spinal TB is relatively low, and spinal TB in children is even rarer. So, the study only illustrated the efficacy of PEEK cage combined with single-segment instrumentation for the treatment of lumbar TB in children. There are several shortcomings to the present study. Firstly, these include its retrospective design lack of comparison groups, small sample size, and relatively short follow-up time. Therefore, large-sample sizes, and randomized as well as controlled types of studies with increasing breadth should be carried out in order to further assess the validity, safety, and applicability of our findings.

## Conclusions

Posterior-only approach using PEEK cage combined with single-segment instrumentation was found to have provided satisfying outcomes for the treatment of lumbar TB in children. Such procedures can reconstruct spinal stability and mostly retain lumbar mobility with less invasion.

## Abbreviations

PEEK: Polyetheretherketone
VAS: Visual analog scale
ASIA: American Spinal Injury Association
ESR: Erythrocyte sedimentation rate
TB: Tuberculosis
